# Metagenomic Analysis of Samples from Three Bat Species Collected in the Amazon Rain Forest

**DOI:** 10.1128/MRA.01422-18

**Published:** 2019-01-10

**Authors:** Luciano Chaves Franco Filho, Rafael Ribeiro Barata, Jedson Ferreira Cardoso, Janaina Mota de Vasconcelos Massafra, Poliana da Silva Lemos, Livia Medeiros Neves Casseb, Ana Cecilia Ribeiro Cruz, Marcio Roberto Teixeira Nunes

**Affiliations:** a Centro de Inovações Tecnológica, Instituto Evandro Chagas, Ananindeua, Pará, Brazil; b Seção de Arbovirologia e Febres Hemorrágicas, Instituto Evandro Chagas, Ananindeua, Pará, Brazil; c Instituto de Ciências Biológicas, Universidade Federal do Pará (UFPA), Belém, Pará, Brazil; University of Delaware

## Abstract

We report here the sequencing of five microbiome samples collected from different bat species in the Amazon rain forest. All contigs matching virus sequences were assigned to members of the *Retroviridae* family, while the bacterial contigs matched several bacterial species mostly belonging to the *Proteobacteria* phylum.

## ANNOUNCEMENT

Several studies have shown that bats are potential natural reservoirs of various pathogens that cause many serious diseases in animals and humans (1–7). Despite several indications that bats potentially harbor distinct pathogens, little is known about the specifics of bats’ microbial communities in Brazil. Due to the association of pathogen outbreaks with bats, metagenomic studies are an important tool for analyzing the circulating viral diversity among these wild animals. Five individuals of three different species of bats, Desmodus rotundus (samples QR02 and QR03), Carollia perspicillata (samples QR05 and QR07), and Artibeus lituratus (sample QR06), were collected in Viseu, Pará, Brazil (01°11′48ʺS, 46°08′24ʺW), using mist nets between 6:00 p.m. and 2:00 a.m. The collected bats were euthanized by intracardiac injection of a solution containing ketamine (75 mg/kg of body weight) and xylazine (3 mg/kg). The project was approved by the Ethics Committee on the Use of Animals of the Evandro Chagas Institute (CEUA/IEC-031/2014) and the Biodiversity Information and Authorization System (SISBIO-47592-1). From each animal, brain and intestine samples were collected and used to prepare the pools. The viral particles were released from the cells using a stainless bead with a TissueLyser II (Qiagen). Subsequently, the samples were preenriched using 0.45-μm-pore filters and an enzymatic treatment (Benzonase; 25 U/liter). DNA and RNA were extracted using the iPrep PureLink virus kit (Thermo Fisher) following the manufacturer’s guidelines. The extracted RNA and DNA were quantified by the Qubit 2.0 fluorometer, using the Qubit RNA high-sensitivity (HS) assay kit and the Qubit double-stranded DNA (dsDNA) HS assay kit (Thermo Fisher). The RNA samples were subjected to reverse transcription using the cDNA synthesis system kit (Roche, Branford, CT) according to the manufacturer’s guidelines. The cDNA and DNA of brain and intestinal tissues were combined and sequenced as a single sample for each bat.

Three samples were sequenced using the Ion Personal Genome Machine (PGM) platform and applying the 200-bp fragment library through the Ion Xpress Plus fragment library kit and the AB library builder system (Thermo Fisher) according to the manufacturer’s recommendations. After the libraries’ construction, sequencing was performed with the Ion 318 chip kit v.2BC. The other two samples were sequenced using the Illumina HiSeq 2500 platform, the Nextera XT DNA library sample preparation kit (Illumina), and the High Output V4 2 × 100-bp sequencing kit (Illumina).

The generated raw data were prefiltered for Q30 quality, and adapters were removed by the Trim Galore pipeline v.0.4.5 (http://www.bioinformatics.babraham.ac.uk/projects/trim_galore/). For the removal of reads with lengths of less than 100 bp, the Prinseq-lite.pl tool (8) was used. Subsequently, the filtered data were submitted to the IDBA-UD assembler v.1.1.3 (9), which used at least five reads with a minimum size of 200 bp to form the contigs. For the k-mers, the *k*_min_ was 21 and *k*_max_ was 101 (*k* + 10). The assembled contigs were compared to the NCBI nonredundant (nr) protein database using the DIAMOND tool v.0.9.22.123 (10) with an E value of 0.00001 (11).

We found viral contigs in four of the five samples analyzed; all contigs belong to the *Retroviridae* family and most of them to the *Betaretrovirus* genus, and in two samples, we found contigs which belong to the *Gammaretrovirus* genus (Table 1). The retroviruses found in all the samples are considered endogenous retroviruses. Furthermore, we found 1,956 contigs with matches assigned to several bacterial species (Table 1). Samples QR02 and QR03 had the largest number of contigs, 533 and 918, respectively, and the main and most abundant bacterial phylum identified was *Proteobacteria*. The phylum *Firmicutes* was the second most abundant in samples QR07 and QR02, whereas *Chlamydiae* was the second in QR03 and *Bacteroidetes* was the second in QR05 (Table 1). The diversity among the samples was quite similar, and the species found are also present in humans, such as Escherichia coli, Salmonella enterica, and the nonfermenting bacterium Acinetobacter baumannii (12, 13).

**TABLE 1:** Sequencing information for each sample

	Value for sample:				
Sequencing information	QR02	QR03	QR05	QR06	QR07
General information					
Sequencing platform	Ion Torrent	Illumina	Ion Torrent	Ion Torrent	Illumina
DNA/cDNA input (ng/μl)	50	1	50	50	1
Total reads (no.)	1,409,806	64,241,920	987,600	258,444	16,881,276
Mapped reads (no.)	106,238	5,872,855	87,632	26,065	1,152,193
Total contigs (no.)	5,132	57,581	3,381	438	12,678
Viral contigs (no.)	1	12	0	5	4
Bacterial contigs (no.)	533	918	215	6	287
Viral matches (no.)					
*Betaretrovirus*	1	8	0	3	4
*Gammaretrovirus*	0	4	0	2	0
Bacterial matches (no.)					
*Fusobacteria*	0	7	0	0	0
*Spirochaetes*	2	9	0	0	0
*Actinobacteria*	3	21	0	2	4
*Firmicutes*	190	97	26	0	51
*Bacteroidetes*	69	128	66	0	45
*Chlamydiae*	37	162	17	0	33
*Proteobacteria*	232	494	106	4	154

### Data availability.

The read data sets of samples QR02, QR03, QR05, QR06, and QR07 have been submitted to the SRA under the accession numbers SRR7537348, SRR7895425, SRR7537349, SRR7537347, and SRR7895424, respectively.
